# Latent curing systems stabilized by reaction equilibrium in homogeneous mixtures of benzoxazine and amine

**DOI:** 10.1038/srep38584

**Published:** 2016-12-05

**Authors:** Jun Wang, Ya Zhen Xu, Ya Fei Fu, Xiang Dong Liu

**Affiliations:** 1 Key Laboratory of Advanced Textile Materials and Manufacturing Technology, Ministry of Education, College of Materials and Textile, Zhejiang Sci-Tech University, Xiasha Higher Education Zone, Hangzhou 310018, P.R. China

## Abstract

Latent curing systems are widely used in industrial thermosets in applications such as adhesion, coating, and composites. Despite many attempts to improve the practicality of this dormant reaction system, the majority of commercially available latent products still use particulate hardeners or liquid compounds with blocked active groups. These formulations generally lack fluidity or rapid reaction characteristics and thus are problematic in some industry applications. Here we describe a novel concept that stabilizes highly reactive benzoxazine/amine mixtures by reaction equilibrium. These new latent benzoxazine curing systems have a long storable lifetime but very short gel time at 150 °C. The reversible reaction between benzoxazine and amine is further demonstrated by FT-IR spectral measurements and rheological experiments, and it is shown that the overall characteristics of the latent system are promising for many industrial applications.

When liquid reactants are mixed homogeneously, chemical reactions occur at a characteristic reaction rate depending on reactant concentrations, activation energy, temperature, and other factors. However, for many industrial polymerizations, this kinetic rule is a problem from chemical engineering and processing perspectives. The adverse examples can be found in a variety of traditional industries such as adhesion, coating, and composites and in numerous modern technologies such as reaction injection molding, resin transfer molding, and 3D printing^1^. These applications demand good fluidity, low viscosity, and sufficient surface wettability for improving formability in the molding and processing stages^2^; therefore the reactive mixtures should be completely inert under storage conditions for a long time, generally more than three months for their storage and delivery^3^. However, the curing processes of these applications often prefer a rapid curing rate to meet the requirements of high-speed production lines. Combined with another demand that the curing temperature should be as low as possible (ideally at 80 °C) for saving energy and for protecting sensitive parts in automobile and electronic industries, the control of polymerization rate in both storage and curing conditions is very challenging because it is generally out of the range controllable by using suitable reactions with large activation energies.

The use of the latent reaction system, which is dormant in storage conditions (often at room temperature) but rapidly reacts in curing condition (commonly >100 °C), is one of the most powerful methods used to solve this dilemma in the polymer industries^4^,^5^,^6^,^7^,^8^. Specifically, latent epoxide formulations have led to profound developments in the applications of epoxy resins relevant for adhesion, coating, and composites^9^,^10^. Traditional epoxy formulations have two basic components, epoxide and hardener reagent (e.g., multivalent amines) that must be stored separately and mixed immediately prior to use^3^. The latent curing systems are pre-mixed formulations containing both epoxide and hardener reagent, and displaying advantages of simple usage and controllable processing window^3^. The use of particulate hardeners (including reactive emulsions) mixed with the liquid epoxide (Fig. 1a)^11^,^12^,^13^,^14^ has been the most successful strategy for the preparation of latent epoxy curing system. For example, dicyandiamide (DICY) powders (particle size smaller than ten micrometers) that are nearly insoluble in epoxy compounds at room temperature have been widely used as a thermally latent curing reagent for epoxy resins^15^. Actually, the reactions of DICY and epoxide are effectively suppressed at the solid DICY surface. However, the micro-particles of hardeners have adverse effects on the fluidity and the viscosity of the curing mixture^16^. For this reason, a number of strategies have been tried for establishing a homogeneous system with the desired curing latency. The blocking of the active groups (such as amines, thiols, and phenols) of hardeners with thermally removable groups (Fig. 1b)^17^,^18^,^19^,^20^,^21^,^22^,^23^ is the main approach for achieving the liquid latent system. Sometimes, these methods may induce sluggish liberation of the active groups, resulting in slow curing processes that are undesirable in commercial uses. Despite the many published reports, commercial formulations of the latent epoxy systems are much less developed. Similarly, to date, only few strategies are available for the applications of other types of polymers such as polyurethane^24^. Development of new concepts for the preparation of latent curing systems having both good stability at storage condition and rapid curing rate at mild temperatures is still challenging.

Polybenzoxazine is a newly developed thermosetting resin which has rich molecular design flexibility^25^,^26^,^27^,^28^,^29^,^30^. Recently, we have demonstrated that benzoxazines react rapidly with amines upon heating at 120 °C, with the curing reaction mechanism involving several reversible reactions^31^. Herein, the reaction of benzoxazines with amines is extended to create a new latent curing system. At the outset of our investigations, we envisioned an innovative concept for a latent curing system based on reaction equilibrium in a homogeneous liquid. This strategy differs fundamentally from the blocked hardeners proceeding to release active groups via thermal deprotection. The reversible reaction of benzoxazine and amine with the resulting intermediate polymer (IP) leads to reaction equilibrium and results in a stable viscosity in room temperature for a long time; however, the reaction equilibrium would be broken by heating and induce a rapid curing (Figs 1c and 2). Theoretically, the precipitation of the resulting polymer network separated from the solution phase is an important factor for promoting the disequilibrium.

## Results and Discussion

**Figure 1:**
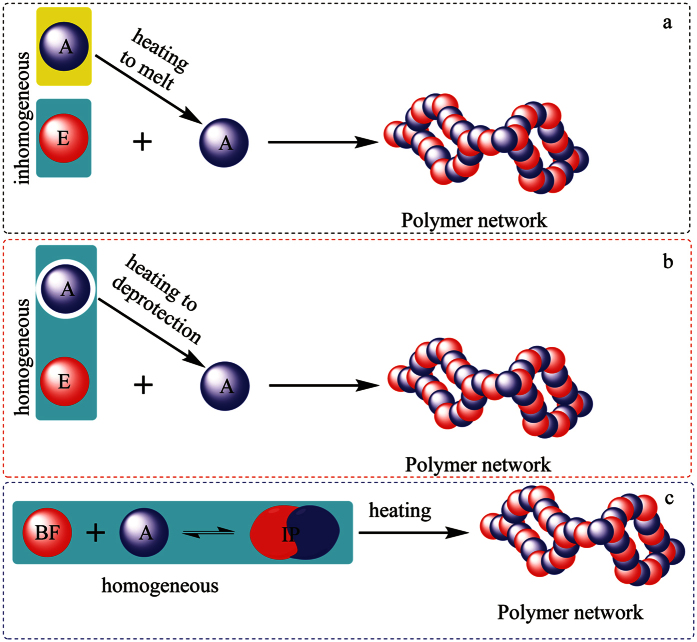
Schematic representations of latent curing systems stabilized by (**a**) particulate surface, (**b**) protective group, and (**c**) reaction equilibrium.

**Figure 3:**
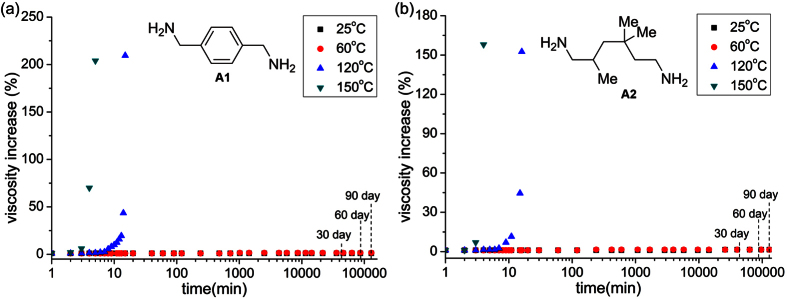
Time dependence of viscosity of reactive mixtures A1/BF (**a**) and A2/BF (**b**) stored at 25 °C, 60 °C, 120 °C, and 150 °C.

To observe the stability of the mixture containing benzoxazine and amine, we initially investigated curing systems of bisphenol-F-benzoxazine (BF) with two amines, m-xylylenediamine (A1) and trimethylhexamethylenediamine (A2). When the solutions of the two mixtures solved in dimethyl formamide (DMF) were stored at 25 °C, their viscosity remained almost constant for a long term (Fig. 3a and b), but increased rapidly over a few minutes after heating at 150 °C. Indeed, despite being stored for one year at 25 °C, the viscosities of the two curing mixtures did not reach the double value. Table S1 presents the latent curing characteristics of the liquid mixtures, including the statistical gel times cured at 120 °C or 150 °C, and the viscosity values stored at 25 °C and 60 °C. Certainly, these parameters of the mixture solutions are suitable for the use as a latent curing system. We have measured the activation energies of the reactions of 6,6′-(propane-2,2-diyl)bis-(3-phenyl-3,4-dihydro-2H-1,3-benzoxazine) with A1 and A2, and found that these energies are equal 75 KJ/mol and 59 KJ/mol, respectively^31^. It is obvious that the stability of the mixture solutions stored at 25 °C and the high curing rates at 150 °C are inconsistent with the Arrhenius equation. The BF/amine mixtures are homogeneous and transparent (Figure S3), implying a new stabilization mechanism different from that shown in Fig. 1a.

The differential scanning calorimetry (DSC) data of the two mixture solutions at the heating rate of 10 °C/min are shown in Figure S1 and Table S2. The BF/amine mixtures showed DSC characteristics that were quite different from those of pure BF. The addition of the amines results in two exothermic peaks at low (<150 °C), and elevated (>180 °C) temperatures, suggesting that at least two reaction mechanisms are present in the curing process. The curing enthalpy values of these DSC peaks were approximately 40 J/g and 13 J/g, respectively, indicating that the reactions of BF with the amines are slightly exothermic in contrast to the large enthalpy value of the thermally induced ring-opening polymerization of bulk BF.

**Figure 4:**
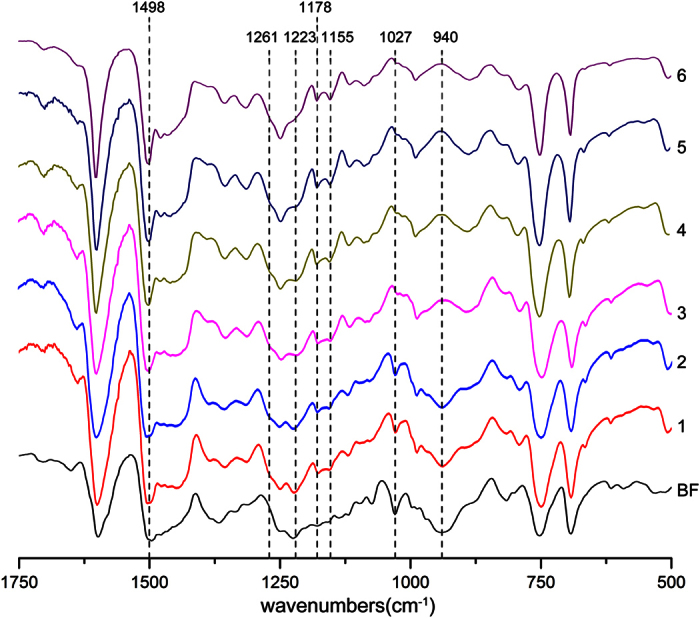
FT-IR spectra of the A1/BF mixture (1) stored at 25 °C for (2) 4 h, (3) 1 day, (4) 5 day, (5) 15 day, and (6) 35 day.

Furthermore, FT-IR measurements of the A1/BF system indicated a prolonged storability at 25 °C (Fig. 4 and Table S3). The oxazine ring of BF showed three characteristic peaks at 940 cm^−1^, 1027 cm^-1^, and 1223 cm^−1^, assigned to the ring symmetric and anti-symmetric stretching of the C-O-C bond^31^,^32^,^33^,^34^,^35^. When the mixture was stored at 25 °C, the three peaks were reduced clearly in the first day, but then become almost constant. These results not only demonstrated the ring-opening reaction of BF but also suggested that the reactive mixture may exhibit a reaction equilibrium. When the curing temperature was fixed at 150 °C, the characteristic peaks of the oxazine rings completely disappeared and characteristic peaks assigned to new chemical structures such as tetra-substituted benzene (1450–1480 cm^−1^)^34^,^35^, significantly increased (Figure S2). This FT-IR spectroscopic evidence suggests that the reaction equilibrium is broken by heating and new polymers are produced.

**Figure 5:**
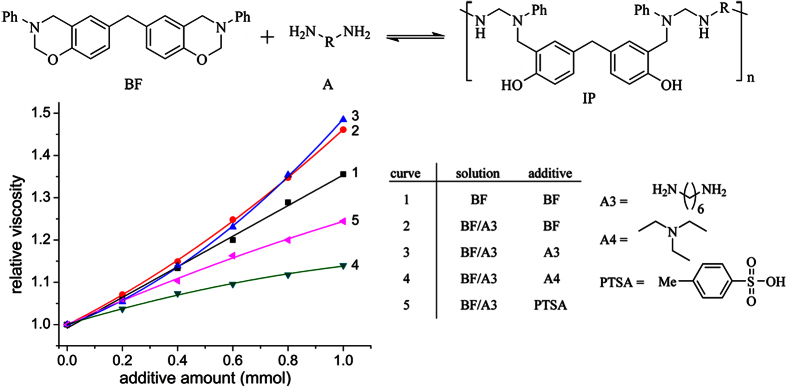
Viscosity change of the BF/A3 mixture solution with various additives.

Measurement of the viscosity of different BF/amine systems enables the observation of the reaction progress and therefore indirectly reflects the reaction equilibrium. Using a rheometer, the BF/amine mixture solutions were isothermally heated at 25 °C, and the rheological data were recorded (Fig. 5). The concentration dependence of the viscosity of polymer solutions is usually represented by the Huggins equation (1).


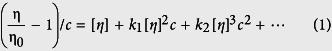


where c is the solution concentration, η is the dynamic viscosity of the solution, η_0_ is the dynamic viscosity of the solvent, [η] is the intrinsic viscosity, and *k*_*1*_ and *k*_*2*_ are the Huggins constants. Because the value of c is sufficiently small for the higher-order terms to be negligible, the Huggins equation can be simplified to,


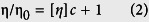


Actually, the rheological results for the BF solution were in good agreement with equation (2), showing that the relative viscosity increases almost linearly with increasing BF concentration (Fig. 5, line 1). However, when a small amounts of BF or hexamethylenediamine (A3) were increasingly added to the BF/A3 mixture solution, the relationship (Fig. 5, curves 2 and 3) between the relative viscosity and the increased solute concentration upward deviated from the straight line (line 1). These phenomena are explained by the increase of the intrinsic viscosity, [η]. If the mixture of BF and A3 react following the mechanism shown in Fig. 1c, the increase of BF or A3 can shift the equilibrium to the right, resulting in an increased polymerization degree of the oligomer adduct. Whereas the addition of p-toluene sulfonic acid (PTSA, consuming some A3 by neutralization with A3) to the BF/A3 mixture drive the reaction equilibrium to the left, causing the polymerization degree of the oligomer adduct to decrease and resulting in the downward curve (curve 5). Because triethylamine (A4) can decrease intermolecular bonds (phenol with amines) between the IP chains, the addition of A4 results in a more downward curve (curve 4) than curve 5.

**Figure 6:**
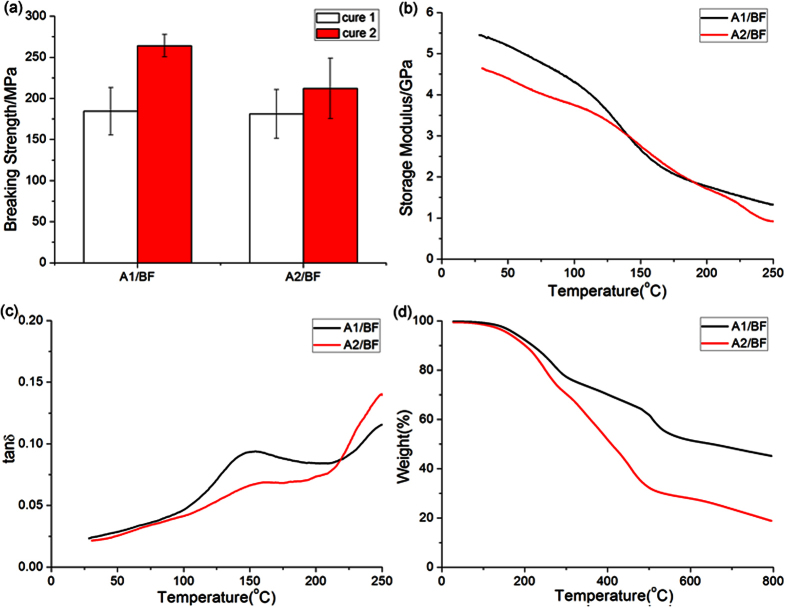
Material properties of cured resins: breaking strength (**a**), temperature dependence of the storage modulus (**b**) and tan δ (**c**), and TGA thermograms (**d**). (Cure conditions: (1) 120 °C/2 h, 150 °C/2 h; (2) 120 °C/2 h, 150 °C/2 h, 180 °C/2 h).

The mechanical and thermal properties of the cured resins are of key importance for their use as a thermoset. Figure 6a and Table 1 summarize the breaking tensile results of the BF/amine mixtures bonding in two aluminum sheets. The DSC results showed that the ring-opening polymerization of the pure BF monomer generally happens after heating to 200 °C. Therefore, the curing of the pure BF monomer at 150 °C produce only a small breaking tensile strength of 44.53 MPa. However, the addition of amine A1 or A2 led to a breaking tensile strength greater than 180 MPa, reaching more than 70% of that of the resin cured at 180 °C. These results demonstrated that the addition of the amines promote the curing process of benzoxazine. Because A1 have the benzene ring structure, the cured BF/A1 mixture showed a stronger breaking strength but a lower stretch rate than that of the cured BF/A2 mixture.

Furthermore, the latent mixtures were cured to composite samples with standard filter paper (average pore size is 18 μm) as a reinforcement filler to reduce the errors generated in the forming process, and were evaluated using dynamic mechanical analyses (DMA) and thermal gravity analysis (TGA). As shown in Fig. 6b, DMA experiments showed that the amine-cured BF resins exhibit high storage modulus values ranging from 4.5 to 5.5 GPa, implying that a highly cross-linked network was formed. The peak that appeared at 150 °C in the tan δ data (Fig. 6c) is in good agreement with optical images of the cured resins (Figure S4), suggesting that a homogeneous polymer network was obtained in this curing process.

Figure 6d shows the TGA profiles and Table 2 provides the important data collected from the TGA thermograms. The TGA curves indicated that all thermal degradation temperatures are higher than 150 °C, may be due to the excessive amine. The carbon residue rate of the cured pure BF resin is 46%, whereas the cured BF/A1 and BF/A2 resins are 51% and 24%, respectively. The aromatic amine A1 showed a positive effect on the carbon residue rate due to the increased amount of aromatic rings in the cured resin, but the aliphatic amine A2 had a negative effect that is explained by the decomposition of the aliphatic chains at high temperatures.

## Conclusion

In conclusion, we described a new thermal latent curing concept basing on the stabilization caused by reaction equilibrium in a homogeneous solution of benzoxazine and amine. FT-IR spectral measurements and rheological experiments suggest that the reaction between BF and amine is reversible and that the reaction equilibrium stabilizes the curing mixture for a long time at low temperature. By heating up to 120 °C, the reaction equilibrium is broken by the formation of a polymer network, resulting in a rapid cure to thermoset resin. These results represent the first demonstration of a latent curing system based on reaction equilibrium in the homogeneous liquid phase. The stability at room temperature, the reactivity induced by heating, and the material characteristics make the new latent reaction concept highly interesting for various applications such as coatings, adhesives, composites, and healable materials.

## Methods

### Materials

Bisphenol F benzoxazine (BF) solution in butanone (75 wt%) was purchased from Huntsman investment Co., Ltd (Utah, USA). M-xylylenediamine (A1), trimethylhexamethylenediamine (A2), hexamethylenediamine (A3), triethylamine (A4), P-Toluene Sulfonic Acid (PTSA) were purchased from Aladdin Reagent Co. (Shanghai, China). All other chemicals were purchased from Hangzhou Mike Chemical Agents Co. Ltd (Hangzhou, China).

### Measurements

FI-TR spectra were obtained on a Nicolet 5700 FT-IR plus spectrometer (Nicolet Company, USA) with 32 scans at a 4 cm-1 resolution. The viscosity values were measured using a Brookfield CAP 2000+ viscometer (Shimadzu Co., USA) or a Physica MCrR301 rheometer (Anton paar, Germany). Thermal analyses were carried out by a Differential Scanning Calorimeter (DSC-1, Mettler-Toledo corp., Switzer-land) with a heating rate of 10 °C/min and a nitrogen flow rate of 35 mL/min. The The absorbance data were obtained on 722E Visible Spectrophotometers (Shanghai spectrum, China).Thermal Gravity Analyses (TGA) were performed on a thermogravimetric analyzer (TGA/DSC-1. Mettler-Toledo corp., Switzer-land) at a heating rate of 10 °C/min from 30 °C to 800 °C under a nitrogen atmosphere. Dynamic Mechanical Analyses (DMA) were performed with a dynamic mechanical analyzer (DMA 1, Mettler-Toledo corp., Switzer-land) with a heating rate of 3 °C/min from 30 °C to 250 °C at 1 Hz. The samples of 10 mm width were cut from the cured films with an approximate thickness of 0.6 mm. The mechanical properties of the cured samples bound between two aluminium sheets (bond surface area: 1.0 cm × 2.0 cm; binding thickness: fixed by glass beads of 0.2 mm diameter) were measured by a material tester (HESON HS300C, Shanghai Heson Ltd., China) with a speed of 10 mm/min at room temperature.

### Preparation of BF/amine mixtures and their cured resins

(1) A BF solution in butanone (75 wt%) was dried in a reduced pressure at 100 °C to obtain BF powder (4.34 g, 0.010 mol), mixed with a diamine (A1 or A2, 0.011 mol), dissolved in DMF (5.0 ml), stored at 25 °C, and used for latent tests (including viscosity and gel time) directly. (2) The A4/BF solutions (10 ml) containing 0.20 mmol BF and A4 were gradually added BF, A3, A4, and PTSA by an amount of 0.20 mmol, respectively. Rheological data were measured using the rheometer before the addition of the additives at 25 °C. The rheological measurement and the addition were repeated for five times. (3) BF power (4.34 g, 0.010 mol) and a diamine (A1 or A2, 0.011 mol) were dissolved in chloroform (8 ml) together. Standard filter papers (with an average pore size of 18 μm) were immersed in the mixture solutions for one day, weight up to 150%, clamped using polyimide sheets, dried under vacuum to remove the solvent, and progressively cured at 120, 150, 180 °C for 2 h. The resulted composite samples were subjected to DMA and TGA tests.

## Additional Information

**How to cite this article**: Wang, J. *et al*. Latent curing systems stabilized by reaction equilibrium in homogeneous mixtures of benzoxazine and amine. *Sci. Rep.* **6**, 38584; doi: 10.1038/srep38584 (2016).

**Publisher's note:** Springer Nature remains neutral with regard to jurisdictional claims in published maps and institutional affiliations.

## Supplementary Material

Supporting Information

## Figures and Tables

**Figure 2:**
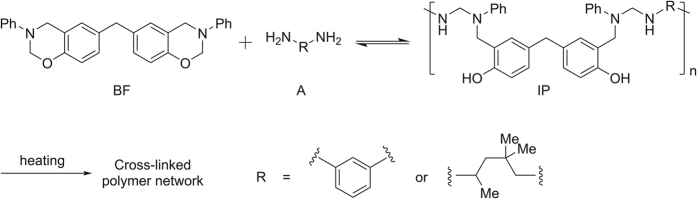
Thermal latent curing system basing on reaction equilibrium.

**Table 1:** The breaking tensile strength and stretch ratio of the cured amine/BF resins.

runs	amine	BF	curing condition	strength (MPa)	stretch (%)
1	A1	BF	120 °C/2 h, 150 °C/2 h	184.40 ± 28.88	1.613 ± 0.167
2	A2	BF	120 °C/2 h, 150 °C/2 h	181.03 ± 29.67	1.790 ± 0.234
3	A1	BF	120 °C/2 h, 150 °C/2 h, 180 °C/2 h	264.13 ± 13.12	1.678 ± 0.178
4	A2	BF	120 °C/2 h, 150 °C/2 h, 180 °C/2 h	212.02 ± 36.68	1.875 ± 0.246

**Table 2:** TGA data selected from the TGA curves (Fig. 4d) of the cured amine/BF resins.

System	T_d5%_ (°C)	T_d10%_ (°C)	T_dmax_ (°C)	CY (%)
BF	195	235	485	46
A1/BF	175	219	507	51
A2/BF	159	201	458	24
