# Supplementary material for: Latent curing systems stabilized by reaction equilibrium in homogeneous mixtures of benzoxazine and amine
Source: Sci Rep. 2016 Dec 5;6:38584. doi: 10.1038/srep38584 (PMC5137143; doi:10.1038/srep38584)
Supplement: Supporting Information [file srep38584-s1.doc]

# Supporting Information

# *Latent curing systems stabilized by reaction equilibrium in homogeneous mixtures of benzoxazine and amine*

**Jun Wang, YaZhen Xu, YaFei Fu, XiangDong Liu***

Key Laboratory of Advanced Textile Materials and Manufacturing Technology, Ministry of Education, College of Materials and Textile, Zhejiang Sci-Tech University, Xiasha Higher Education Zone, Hangzhou 310018, People’s Republic of China

Correspondence to: Xiang Dong, Liu, (E-mail: liuxd@zstu.edu.cn)

Table S1. Gel times and viscosity of the amine/benzoxazine mixtures.

| Mixed system | A1/BF | A2/BF |
| --- | --- | --- |
| Gel time (s)  120 ^o^C | 820±30 | 900±50 |
| 150 ^o^C | 150±10 | 190±30 |
| Viscosity (P) 25 ^o^C  0 day  1 day  30 day  60 day  90 day | 1.500  1.500  1.500  1.500  1.500 | 1.500  1.500  2.250  2.250  2.250 |
| Viscosity (P) 60 ^o^C  0 day  1 day  30 day  60 day  90 day | 1.500  2.250  2.250  2.250  2.250 | 1.500  2.250  2.250  2.250  2.250 |

Table S2. DSC data of the reactive BF/amine mixtures

| **sample** | **Peak 1** | | | **Peak 2** | | |
| --- | --- | --- | --- | --- | --- | --- |
|  | T_0_(^o^C) | T_P_(^o^C) | △H(J/g) | T_0_(^o^C) | T_P_(^o^C) | △H(J/g) |
| **pure BF** | / | / | / | 201.50 | 228.45 | 253.74 |
| **A1/BF** | 70.60 | 130.83 | 43.04 | 185.41 | 215.0 | 13.99 |
| **A2/BF** | 91.40 | 132.34 | 47.41 | 211.17 | 241.51 | 6.79 |
| **A3/BF** | 100.66 | 141.46 | 41.27 | 192.55 | 237.10 | 12.68 |

Table S3. Analyses of the FT-IR peaks in Figure 3.

| Wavenumbers | attribution | change | explanation |
| --- | --- | --- | --- |
| 940 cm^-1^, 1027 cm^-1^, 1223 cm^-1^ | C-O-C in oxazine ring | decrease | the ring-opening reaction of BF by reaction with A1.  the reaction of A1 and BF reaches an equilibrium. |
| 1178 cm^-1^ | C-N | increase slightly |
| 1155 cm^-1^ | C-N-H | increase slightly |
| 1498 cm^-1^ | tri-substituted benzene | decrease slightly |  |
| 1261 cm^-1^ | Ph-OH (C-O-H) | increase | formation of phenolic hydroxyl groups. |


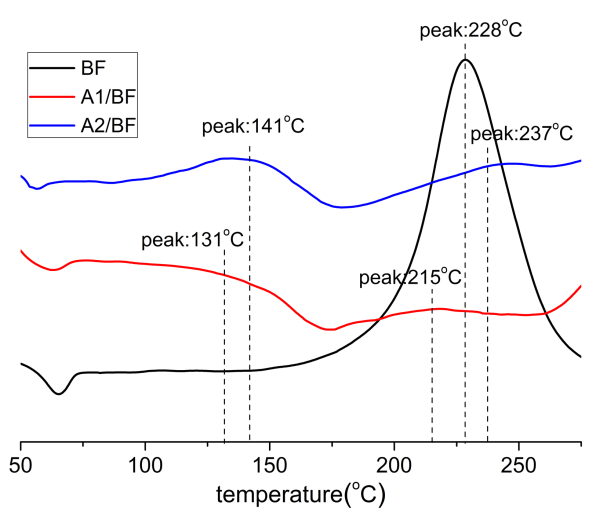


Figure S1. DSC curves for BF without (black) and with amines A1 (red) and A2 (blue).


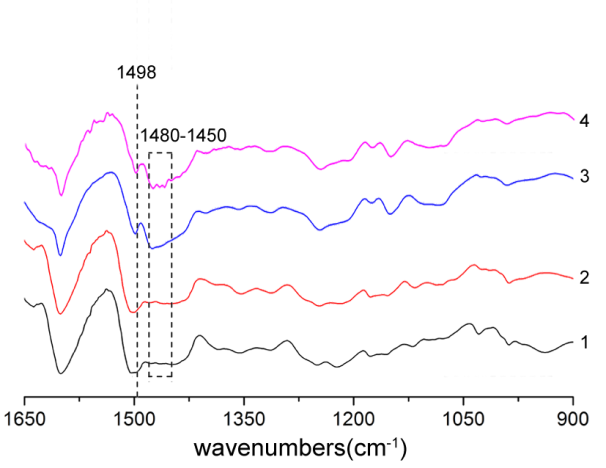


Figure S2. FT-IR spectra of A1/BF mixtures and their cured products. (1) A1/BF mixture, (2) A1/BF mixture stored at 25 ^o^C for one day, (3) cured product of mixture 1, and (4) cured product of mixture 2. (Curing condition: 150 ^o^C/2 h)


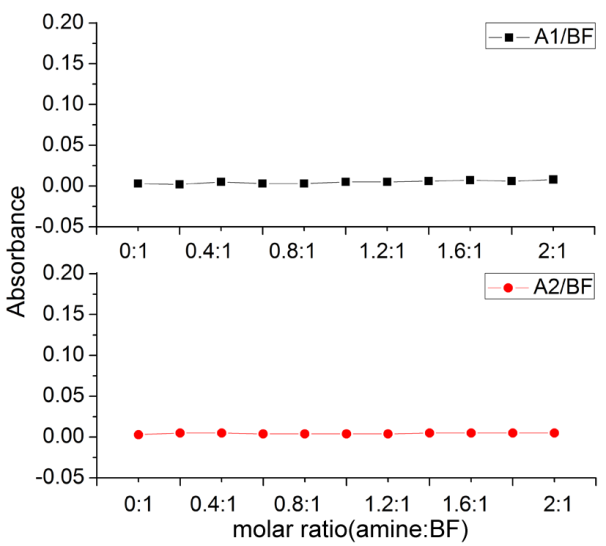


Figure S3. The light absorbance (730 nm) data of the amine/BF mixtures.


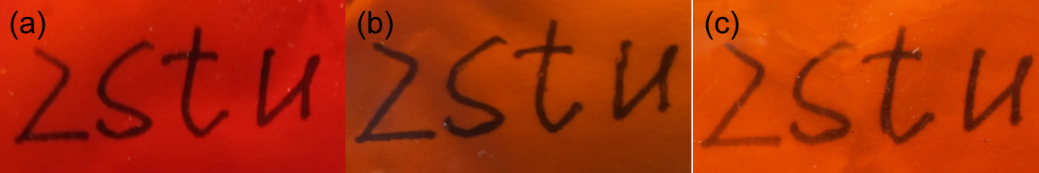


Figure S4. The optical images of the product resins cured from (a) BF, (b) A1/BF, and (c) A2/BF. (Cure conditions: 120 ^o^C/2 h, 150 ^o^C/2 h, 180 ^o^C/2 h)
